# Supplementary material for: Discovery of optimal cell type classification marker genes from single cell RNA sequencing data
Source: BMC Methods. Author manuscript; Available in PMC 2025 Aug 30. (PMC12396544; doi:10.1186/s44330-024-00015-2)
Supplement: Supplementary Fig. 1 — Supplementary Figure 1. Comparing performance of using different BinaryFirst thresholds in NS-Forest v4.0 on specific subclades within human MTG dataset. (A) Hierarchical dendrogram derived in the original human MTG study with labelled and color-coded subclades (https://github.com/AllenInstitute/MOp_taxonomies_ontology/tree/main). (B) Heatmaps of markers from the human middle temporal gyrus (MTG) dataset generated from NS-Forest v4.0 with ‘BinaryFirst_mild’, ‘BinaryFirst_moderate’, and ‘BinaryFirst_high’ thresholds. The regions on the heatmaps highlighted by the orange boxes correspond to the identified markers for the cell types in specific subclades (VIP, PVALB, and L4 subclades) that are known to be more similar to each other and thus, more difficult to distinguish. The colors correspond to the normalized median expression level (log2-transformed counts per million) for the marker gene (rows) in a given cell type cluster (columns), with high expression in red/yellow, and low expression in blue/white. The clusters are ordered according to the hierarchical dendrogram provided in the original study shown in (A). (C) Median On-Target Fraction values within each of the three specific subclades across these three BinaryFirst thresholds. [file NIHMS2104291-supplement-Supplementary_Fig__1.pdf]

A

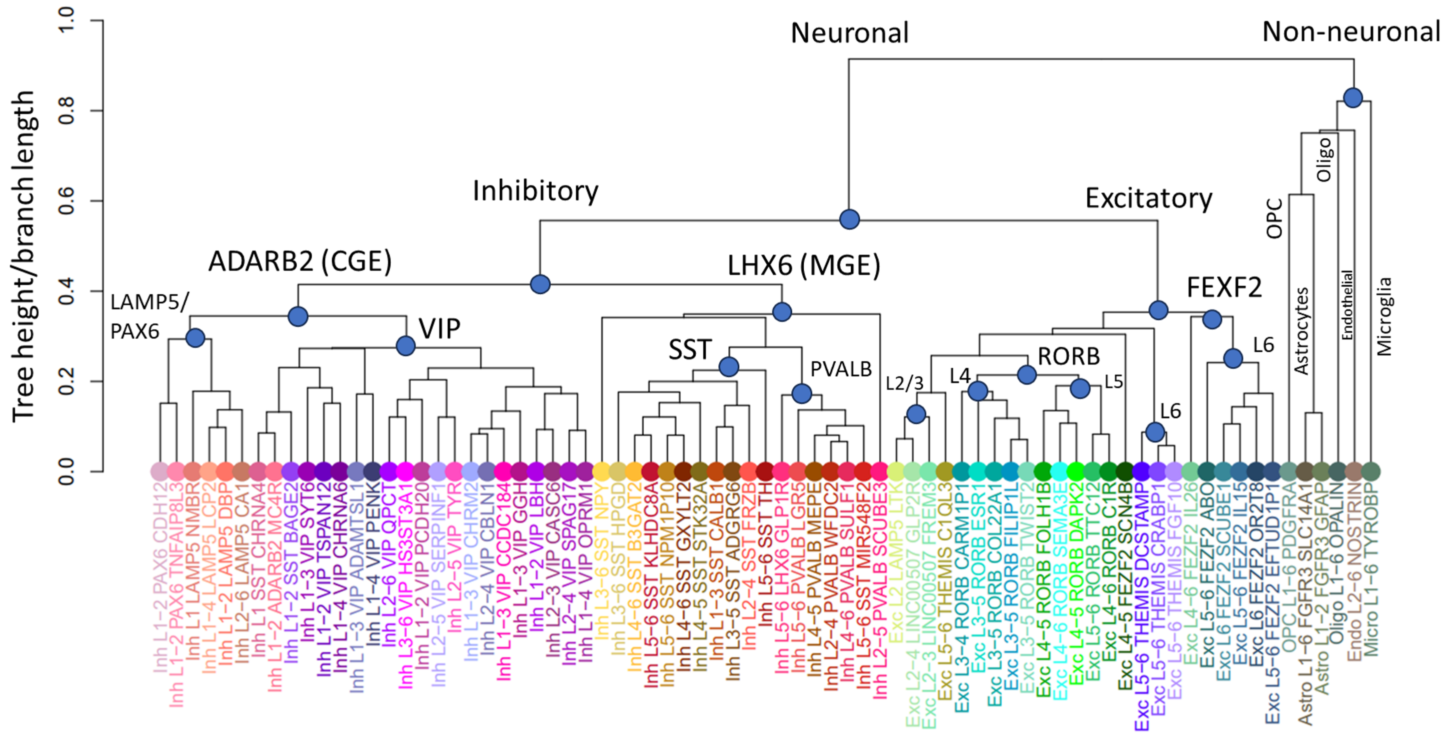

B

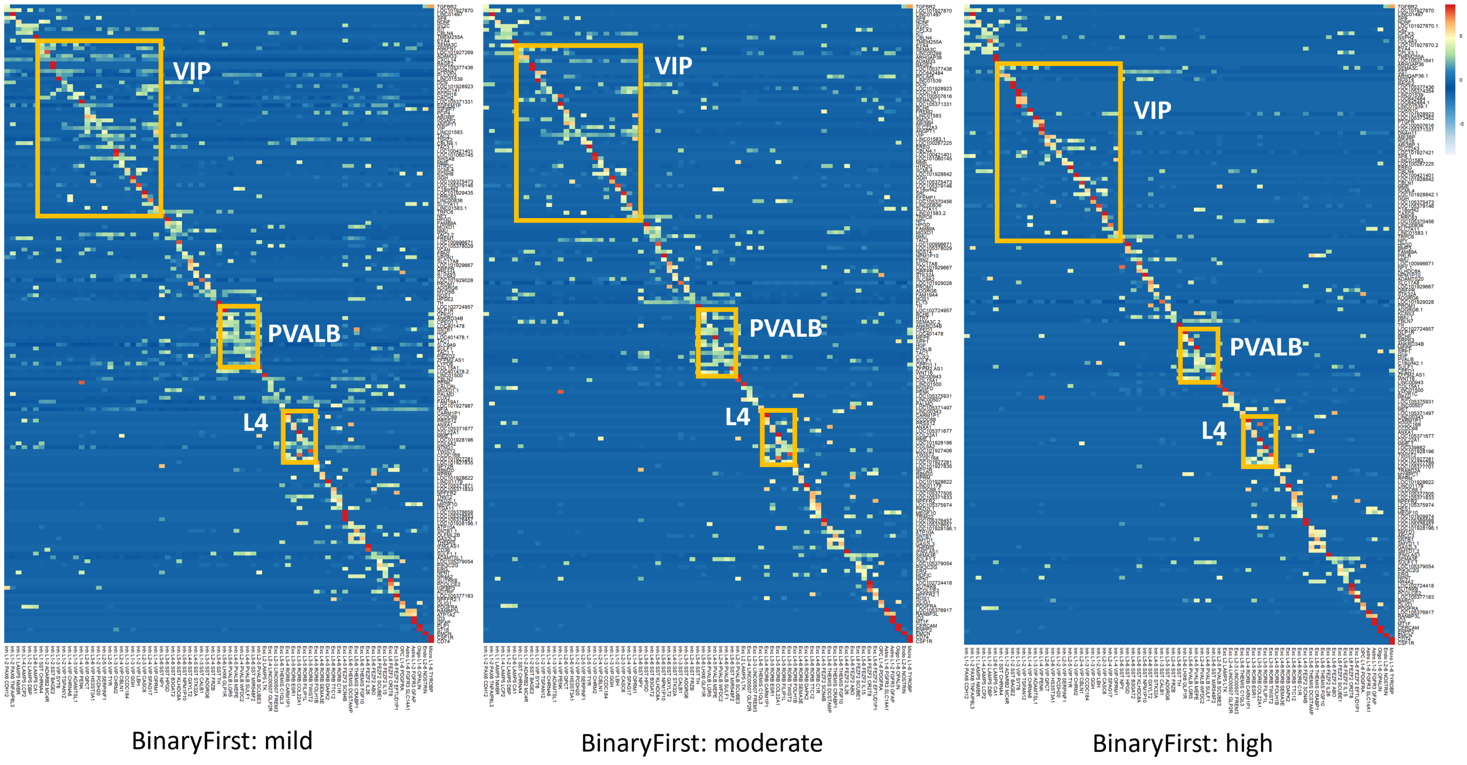

C

Subclade	Approach	Median On-Target Fraction
L4	NS-Forest v2.0/3.9/BinaryFirst: mild	0.368
	BinaryFirst: moderate	0.368
	BinaryFirst: high	0.406
PVALB	NS-Forest v2.0/3.9/BinaryFirst: mild	0.146
	BinaryFirst: moderate	0.153
	BinaryFirst: high	0.286
VIP	NS-Forest v2.0/3.9/BinaryFirst: mild	0.213
	BinaryFirst: moderate	0.296
	BinaryFirst: high	0.385
