# Supplementary material for: Discovery of optimal cell type classification marker genes from single cell RNA sequencing data
Source: BMC Methods. Author manuscript; Available in PMC 2025 Aug 30. (PMC12396544; doi:10.1186/s44330-024-00015-2)
Supplement: Supplementary Fig. 2 — Supplementary Figure 2. Comparison of On-Target Fraction distribution in major subclades of Human MTG dataset across BinaryFirst thresholds. Boxplots comparing the distribution of the On-Target Fraction values within each of the three specific subclades in the human MTG dataset (L4, PVALB, and VIP subclades) across the mild, moderate, and high BinaryFirst thresholds are shown. [file NIHMS2104291-supplement-Supplementary_Fig__2.pdf]

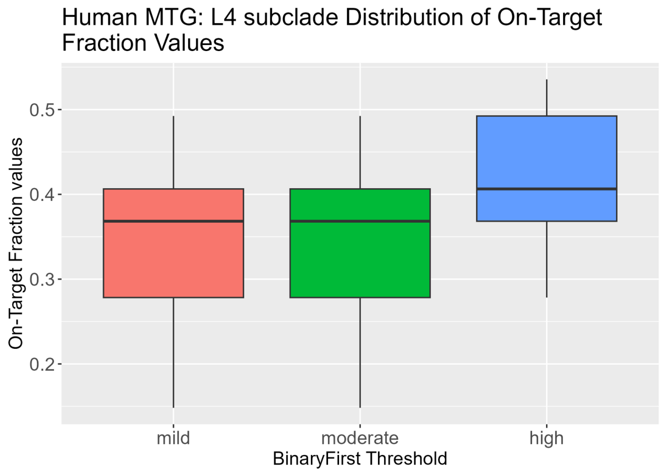

Human MTG: L4 subclade Distribution of On-Target Fraction Values
On-Target Fraction values
0.5
0.4
0.3
0.2
mild
moderate
high
BinaryFirst Threshold

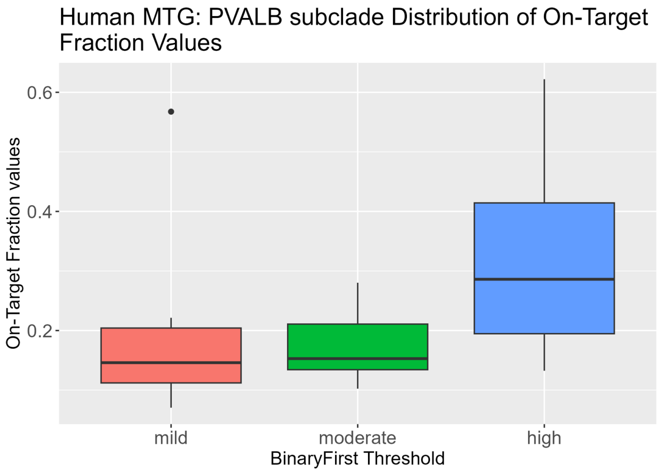

Human MTG: PVALB subclade Distribution of On-Target Fraction Values
On-Target Fraction values
0.6
0.4
0.2
mild
moderate
high
BinaryFirst Threshold

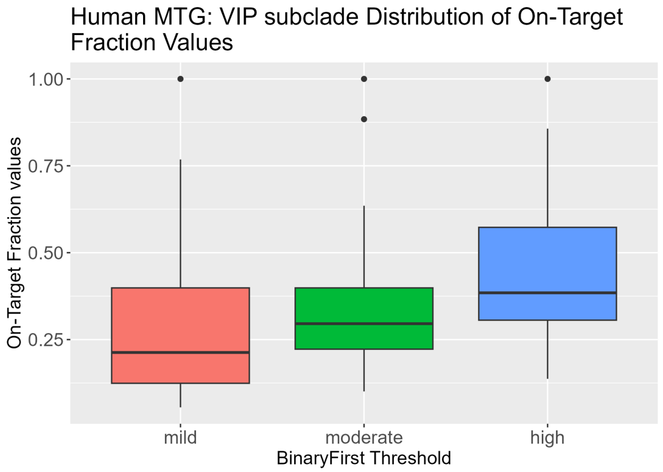

Human MTG: VIP subclade Distribution of On-Target Fraction Values
On-Target Fraction values
1.00
0.75
0.50
0.25
mild
moderate
high
BinaryFirst Threshold
