# Supplementary material for: Discovery of optimal cell type classification marker genes from single cell RNA sequencing data
Source: BMC Methods. Author manuscript; Available in PMC 2025 Aug 30. (PMC12396544; doi:10.1186/s44330-024-00015-2)
Supplement: Supplementary Fig. 3 — Supplementary Figure 3. Additional investigation of mean + 3 standard deviations (SD) BinaryFirst threshold evaluated in the human MTG dataset. (A-B) Heatmaps of NS-Forest marker genes using the BinaryFirst threshold of mean + 3 SD in the human MTG dataset, without and with the VIP, PVALB, and L4 subclades highlighted. (C-D) Performance metrics using the mean + 3 SD threshold in the human MTG dataset, directly comparable with Fig. 3D and Supplementary Fig. 1C. (E) Scatter plots and the best linear relationship fitted for the number of input genes to the random forest (RF) step after BinaryFirst filtering using different thresholds with respect to the On-Target Fraction values per cluster. [file NIHMS2104291-supplement-Supplementary_Fig__3.pdf]

A

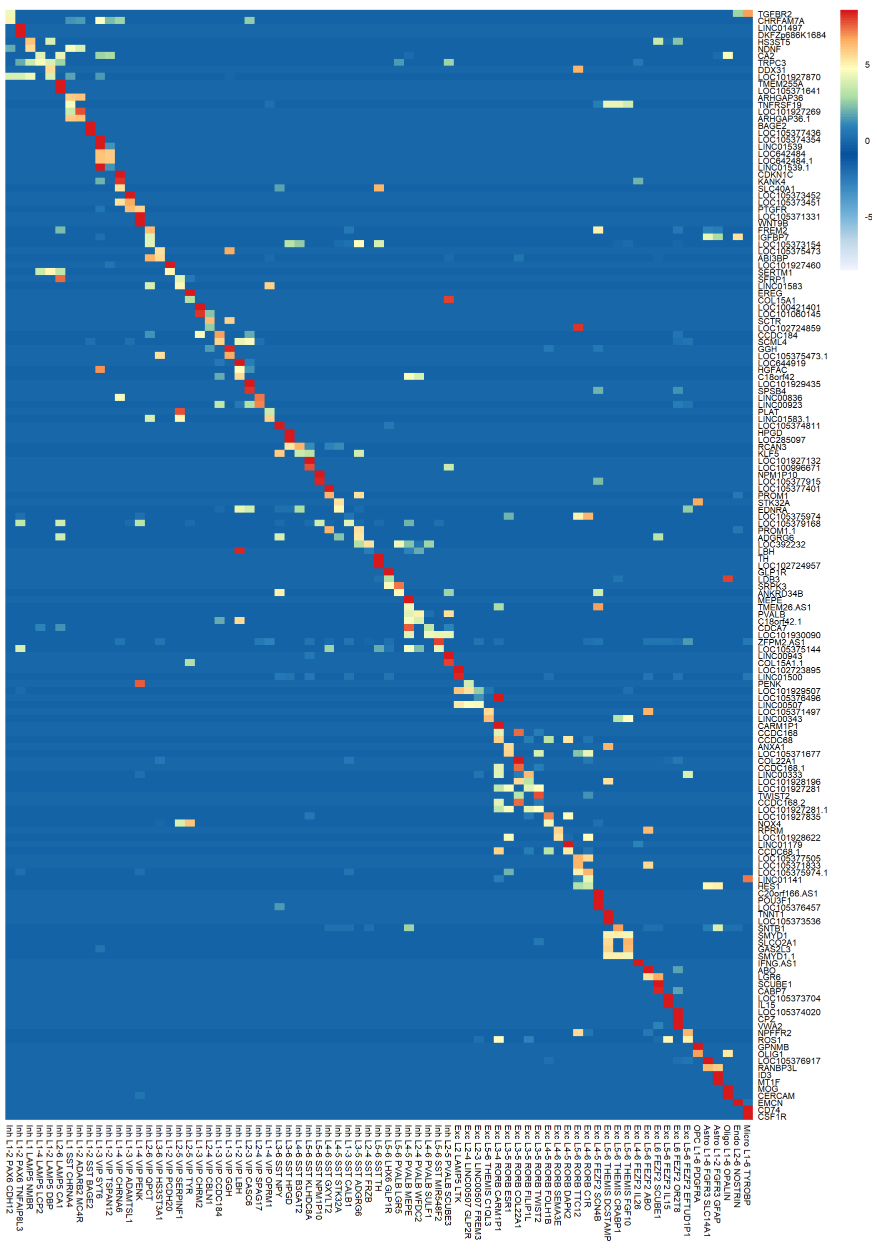

Mean + 3 SD

B

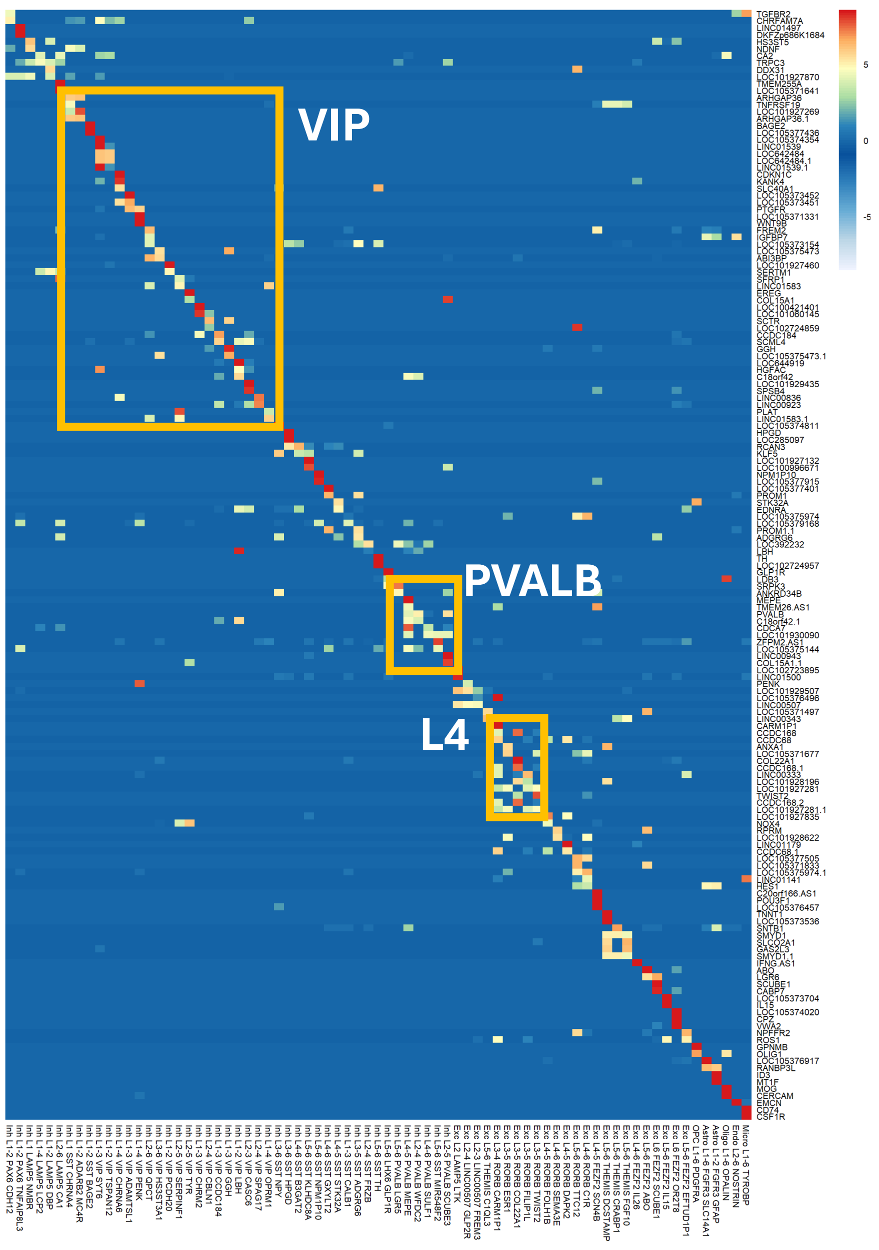

Mean + 3 SD

E

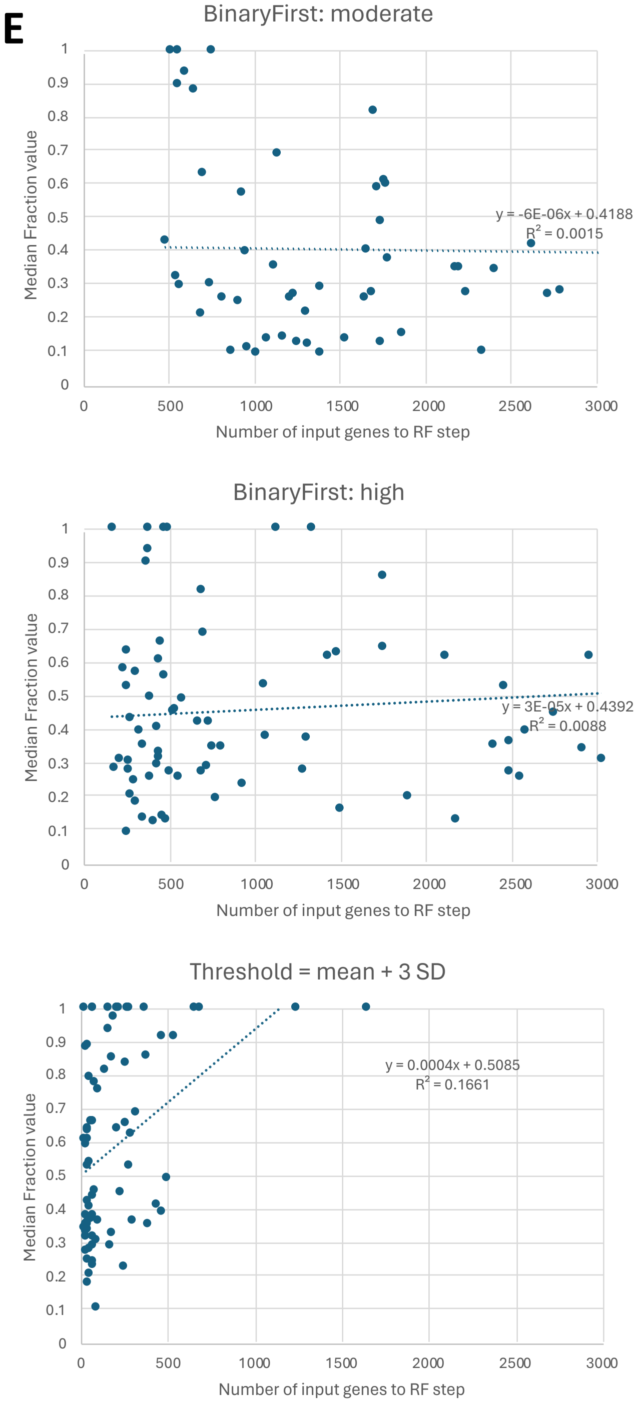

C

	Mean + 3 SD
**Median F-beta**	0.582
**Median PPV**	0.800
**Median On-Target Fraction**	0.543

D

Subclade	Approach	Median on-target fraction
L4	BinaryFirst (mean + 3 SD)	0.3683
PVALB	BinaryFirst (mean + 3 SD)	0.3735
VIP	BinaryFirst (mean + 3 SD)	0.5427
