# Supplementary material for: Discovery of optimal cell type classification marker genes from single cell RNA sequencing data
Source: BMC Methods. Author manuscript; Available in PMC 2025 Aug 30. (PMC12396544; doi:10.1186/s44330-024-00015-2)
Supplement: Supplementary Fig. 4 — Supplementary Figure 4. Evaluating performance of NS-Forest on lung L4 and L3 subclass datasets. (A) Heatmaps of NS-Forest v2.0/v3.9 and v4.0 markers from the L4 and L3 subclasses of the human lung. The colors correspond to the normalized median expression level (log2-transformed counts per million) for the marker gene (rows) in a given cell type cluster (columns), with high expression in red/yellow, and low expression in blue/white. The clusters are ordered according to the hierarchical ordering in the dendrogram generated by the scanpy package (scanpy.tl.dendrogram) using default settings. (B) Comparison of the performance metrics corresponding to the NS-Forest results shown in (A). [file NIHMS2104291-supplement-Supplementary_Fig__4.pdf]

A

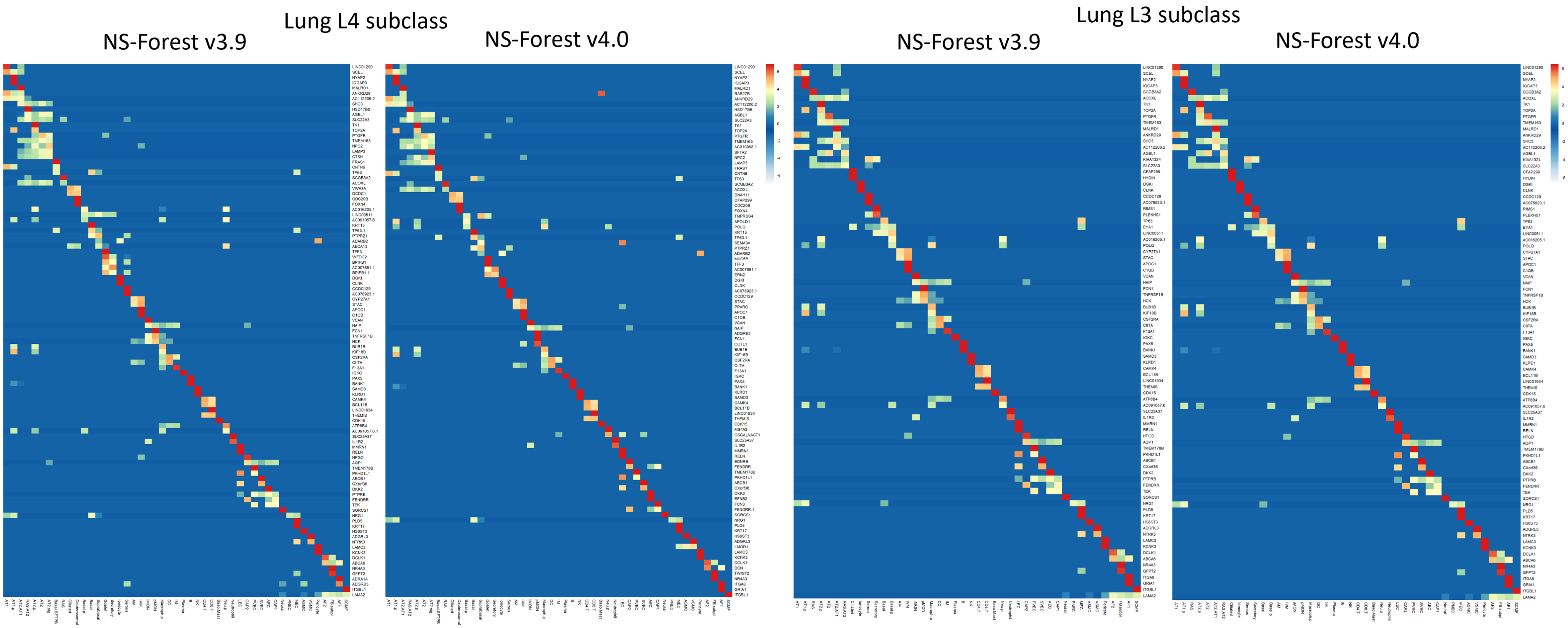

B

	Lung L4 subclass		Lung L3 subclass	
NS-Forest version	*NS-Forest v3.9*	*NS-Forest v4.0*	*NS-Forest v3.9*	*NS-Forest v4.0*
**Median f-beta**	0.676	0.649	0.694	0.692
**Median PPV**	0.859	0.848	0.877	0.873
**Median On-Target Fraction**	0.637	0.746	0.749	0.768
