# Supplementary material for: Discovery of optimal cell type classification marker genes from single cell RNA sequencing data
Source: BMC Methods. Author manuscript; Available in PMC 2025 Aug 30. (PMC12396544; doi:10.1186/s44330-024-00015-2)
Supplement: Supplementary Fig. 5 — Supplementary Figure 5. Distribution of median gene expression per cluster and Binary Expression Score in human MTG, kidney, and lung datasets. First row: histograms of distribution of median gene expression values of genes expressed in all clusters in human MTG, kidney, and lung datasets. X-axis displays the range of median gene expression values in each dataset, and the y-axis displays the frequency of each median gene expression value (log scale). Second row: histograms of distribution of Binary Expression Score values of genes in these three datasets. X-axis ranges from 0 to 1 (representing the possible values the binary expression score can be), and the y-axis displays the frequency of each binary score value (log scale). All distributions are highly right-skewed. [file NIHMS2104291-supplement-Supplementary_Fig__5.pdf]

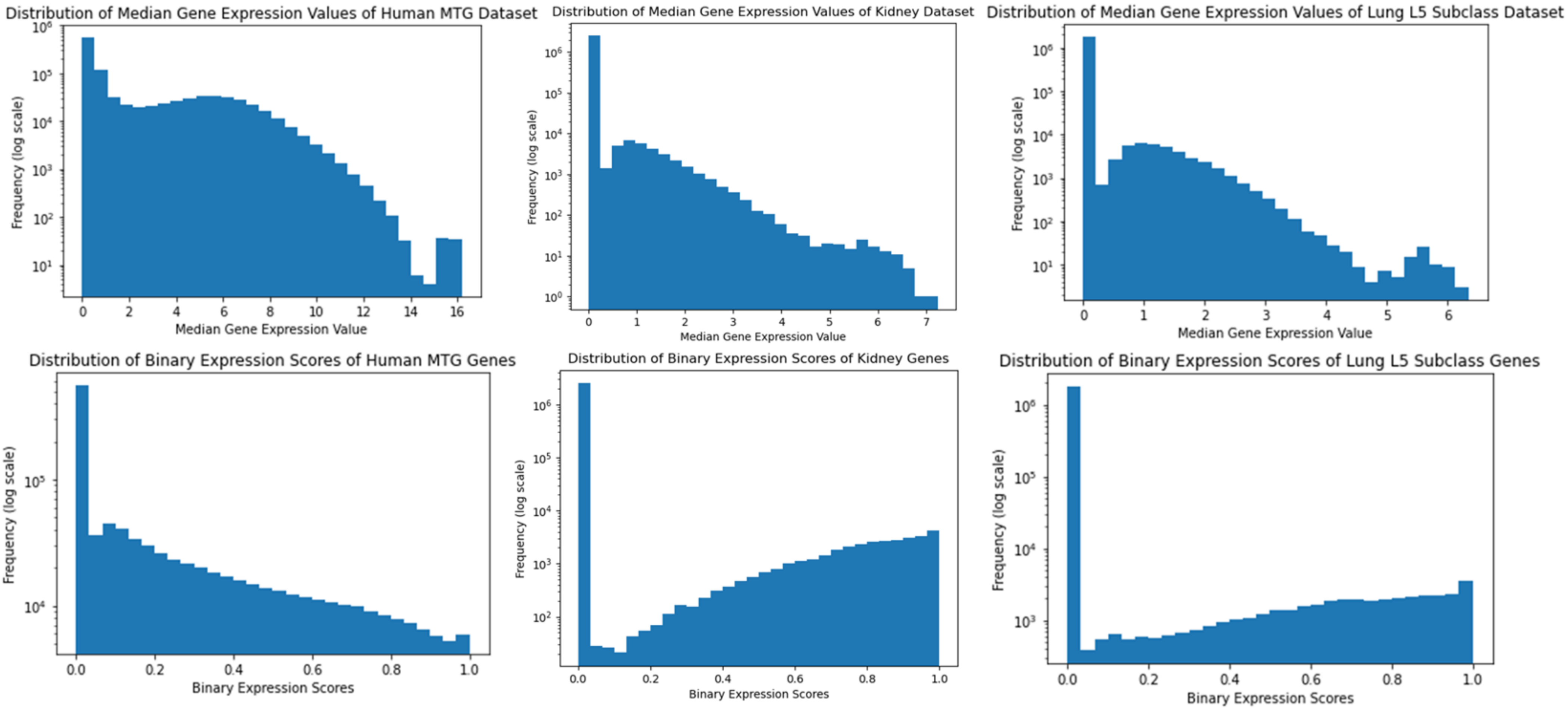

Distribution of Median Gene Expression Values of Human MTG Dataset
Frequency (log scale)
Median Gene Expression Value
Distribution of Median Gene Expression Values of Kidney Dataset
Frequency (log scale)
Median Gene Expression Value
Distribution of Median Gene Expression Values of Lung L5 Subclass Dataset
Frequency (log scale)
Median Gene Expression Value
Distribution of Binary Expression Scores of Human MTG Genes
Frequency (log scale)
Binary Expression Scores
Distribution of Binary Expression Scores of Kidney Genes
Frequency (log scale)
Binary Expression Scores
Distribution of Binary Expression Scores of Lung L5 Subclass Genes
Frequency (log scale)
Binary Expression Scores
