# Supplementary figures and images for: Discovery of optimal cell type classification marker genes from single cell RNA sequencing data
Source: BMC Methods. Author manuscript; Available in PMC 2025 Aug 30. (PMC12396544; doi:10.1186/s44330-024-00015-2)

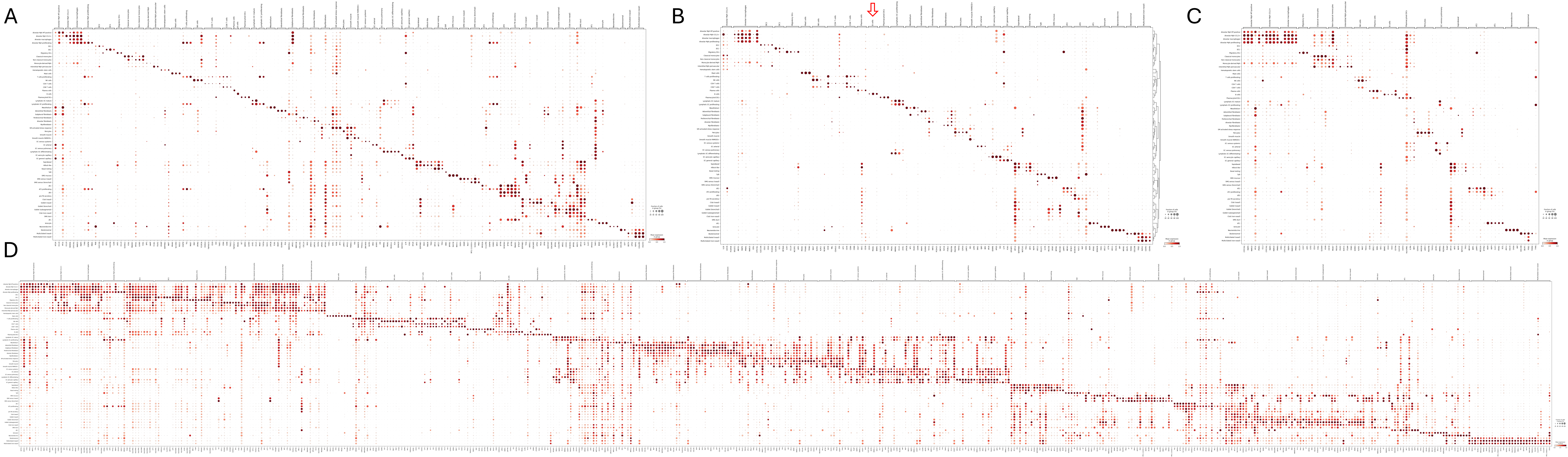

Supplement: Supplementary Fig. 6 — Dotplots of HLCA, CellRef, ASCT + B, and Azimuth marker genes on HLCA core cell types. (A) 162 HLCA markers across 61 HLCA cell types. (B) 115 CellRef markers across 33 HLCA cell types. The highlighted example is where the On-Target Fraction is perfect but recall is low. (C) 80 ASCT + B markers across 18 HLCA cell types. (D) 53 zimuth markers across 56 HLCA cell types. [file NIHMS2104291-supplement-Supplementary_Fig__6.pdf]

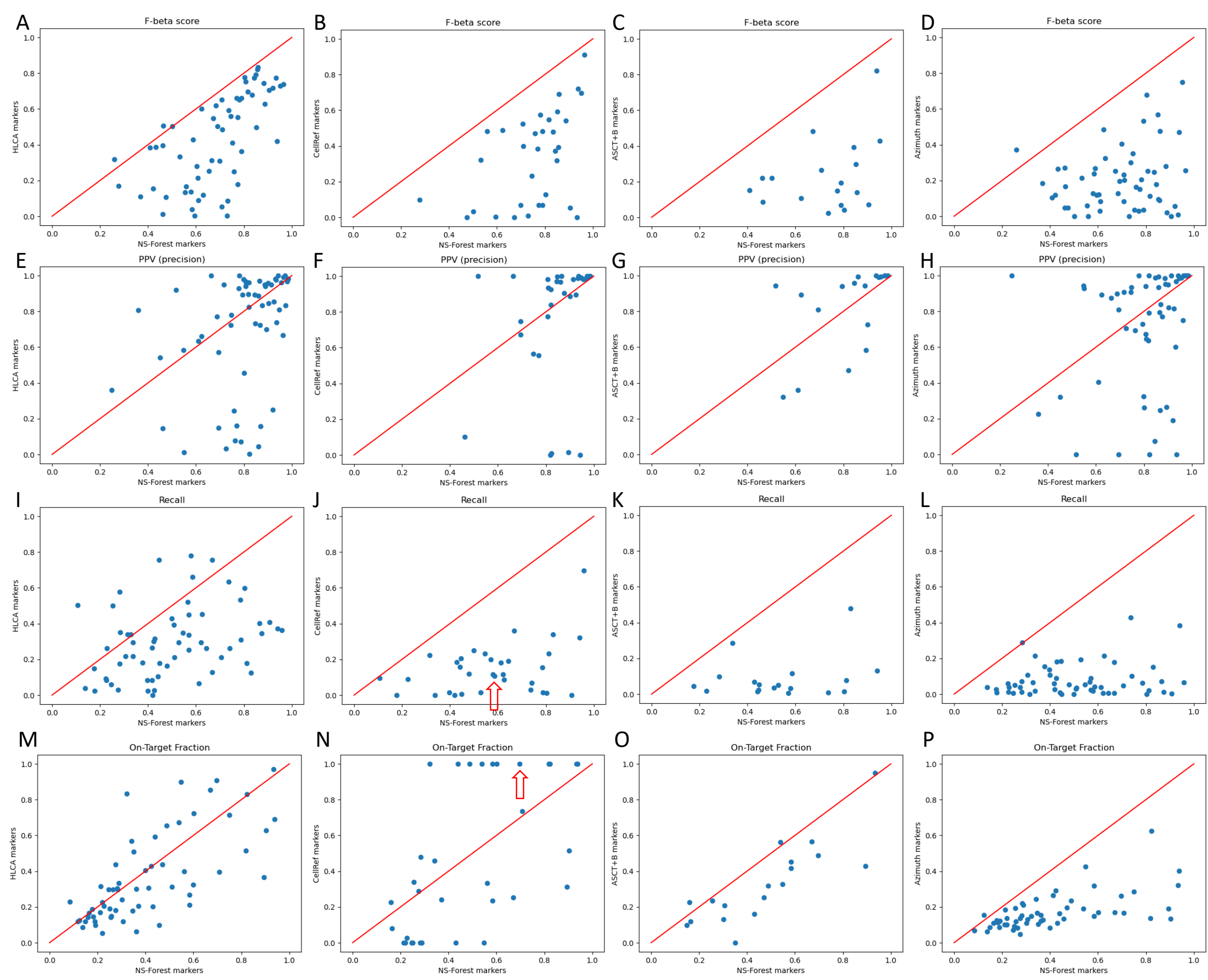

Supplement: Supplementary Fig. 7 — Supplementary Figure 7. NS-Forest consistently outperforms other published lung marker genes in classification performance. (A-D) Scatter plots comparing F-beta scores for each cell type using NS-Forest markers vs. HLCA, CellRef, ASCT + B, and Azimuth markers. (E–H) Scatter plots comparing PPV (precision) for each cell type using NS-Forest markers vs. HLCA, CellRef, ASCT + B, and Azimuth markers. (I-L) Scatter plots comparing recall for each cell type using NS-Forest markers vs. HLCA, CellRef, ASCT + B, and Azimuth markers. (M-P) Scatter plots comparing On-Target Fraction for each cell type using NS-Forest markers vs. HLCA, CellRef, ASCT + B, and Azimuth markers. The highlighted example is where the On-Target Fraction is perfect but recall is low. [file NIHMS2104291-supplement-Supplementary_Fig__7.pdf]

# HLCA Binary Genes Co-expression

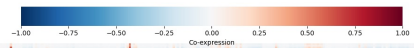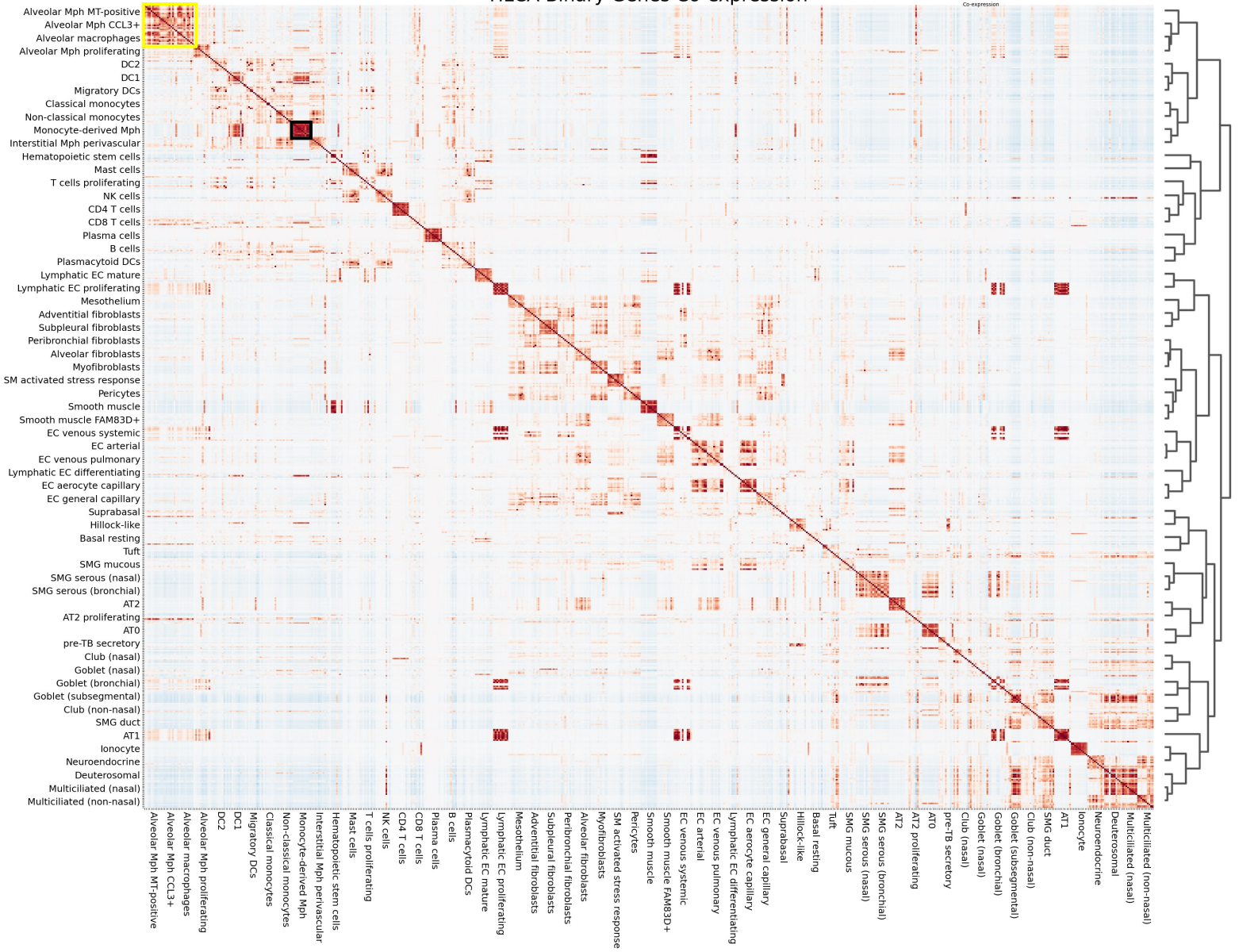

Supplement: Supplementary Fig. 9 — Supplementary Figure 9. Heatmap of co-expression for the binary genes outputted from the NS-Forest algorithm with the HLCA dataset. [file NIHMS2104291-supplement-Supplementary_Fig__9.pdf]
