# Supplementary material for: Discovery of optimal cell type classification marker genes from single cell RNA sequencing data
Source: BMC Methods. Author manuscript; Available in PMC 2025 Aug 30. (PMC12396544; doi:10.1186/s44330-024-00015-2)
Supplement: Supplementary Fig. 8 — Supplementary Figure 8. Simulation results of NS-Forest v2.0/3.9 and v4.0. Heatmap of the number of times that a gene is selected as NS-Forest marker gene in the simulations. By the simulation design, genes 1–5 are true marker genes, genes 6–10 are non-marker genes, and genes 11–100 are null genes (not shown as they won’t be selected by design). [file NIHMS2104291-supplement-Supplementary_Fig__8.pdf]

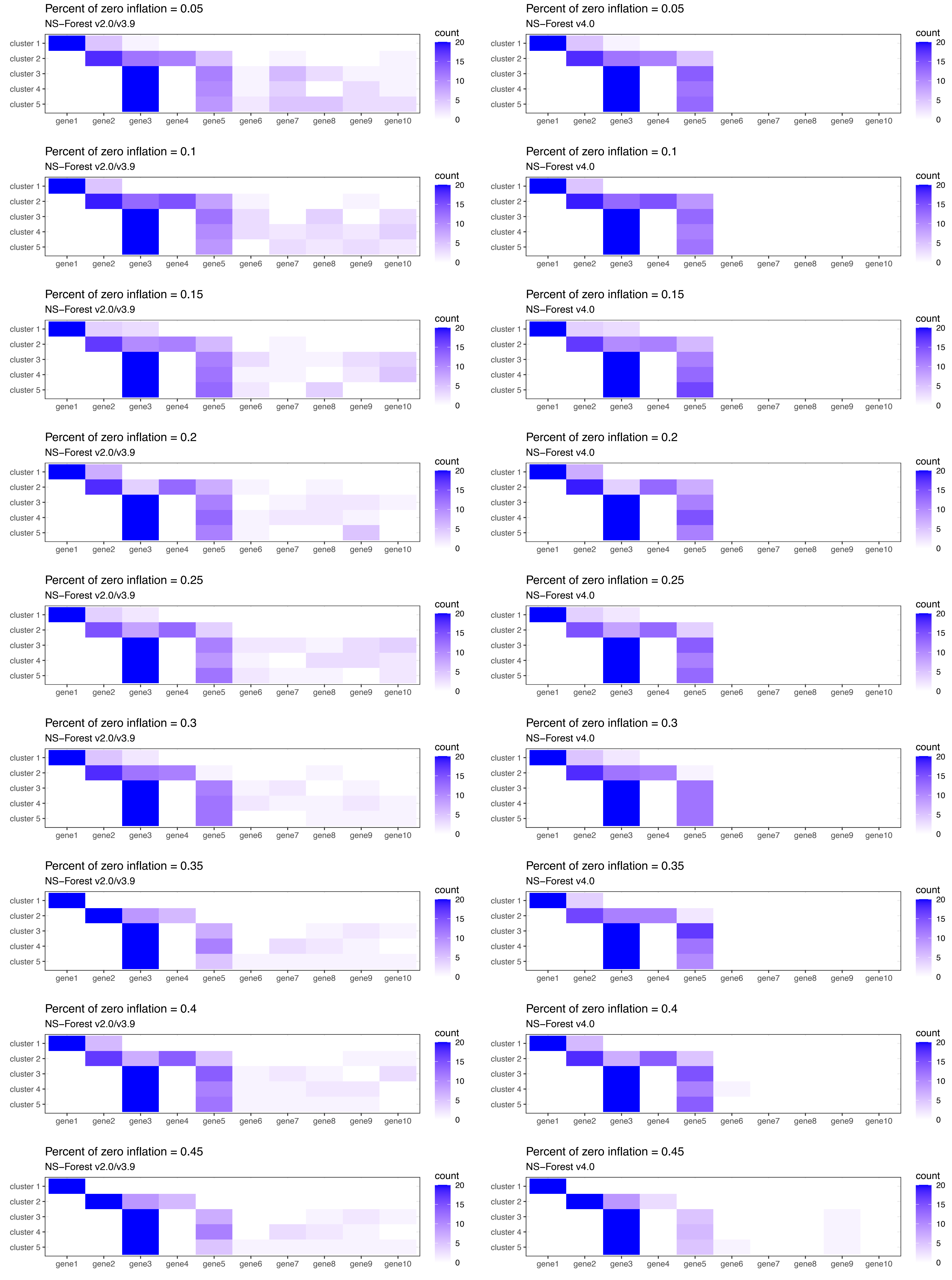
Percent of zero inflation = 0.05
NS−Forest v2.0/v3.9
Percent of zero inflation = 0.05
NS−Forest v4.0
Percent of zero inflation = 0.1
NS−Forest v2.0/v3.9
Percent of zero inflation = 0.1
NS−Forest v4.0
Percent of zero inflation = 0.15
NS−Forest v2.0/v3.9
Percent of zero inflation = 0.15
NS−Forest v4.0
Percent of zero inflation = 0.2
NS−Forest v2.0/v3.9
Percent of zero inflation = 0.2
NS−Forest v4.0
Percent of zero inflation = 0.25
NS−Forest v2.0/v3.9
Percent of zero inflation = 0.25
NS−Forest v4.0
Percent of zero inflation = 0.3
NS−Forest v2.0/v3.9
Percent of zero inflation = 0.3
NS−Forest v4.0
Percent of zero inflation = 0.35
NS−Forest v2.0/v3.9
Percent of zero inflation = 0.35
NS−Forest v4.0
Percent of zero inflation = 0.4
NS−Forest v2.0/v3.9
Percent of zero inflation = 0.4
NS−Forest v4.0
Percent of zero inflation = 0.45
NS−Forest v2.0/v3.9
Percent of zero inflation = 0.45
NS−Forest v4.0
cluster 1
cluster 2
cluster 3
cluster 4
cluster 5
gene1
gene2
gene3
gene4
gene5
gene6
gene7
gene8
gene9
gene10
count
20
15
10
5
0
